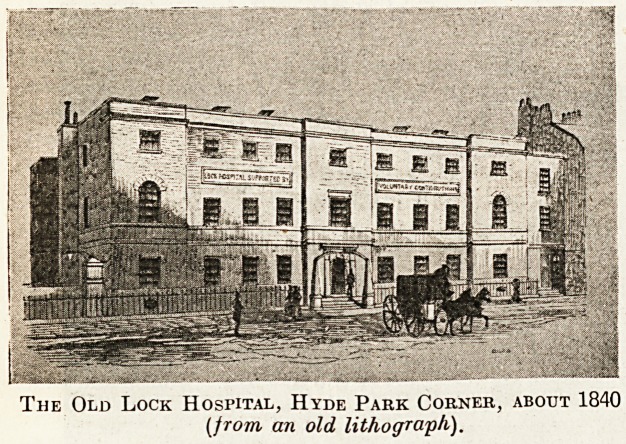# As Depicted by Engraved Views.—III

**Published:** 1920-12-18

**Authors:** 


### THE HISTORY OF HOSPITALS

### AS DEPICTED BY ENGRAVED VIEWS.—III.

OF the old lazar houses very few engraved views are to be found. One of the best known was founded in 1117 in High Street, St. Giles's, by the pious Maud, or Matilda, consort of Henry I., upon the site of which the present parish church was built in 1730, replacing another church which had come to a ruinous condition. In former times High Street was part of the main road proceeding westward from London, and at the hospital door it became a custom to present malefactors, carried to execution at Tyburn, with a great bowl of ale to drink of it as they pleased as their last refreshment in this life. The original endowment of the hospital amounted to £3, a rent charge on Queenhithe, and Henry II. added a further £3 from the exchequer.

### THE OLD LEPER HOUSES.

That the leper houses were, generally speaking, the forerunners of lock hospitals cannot be convincingly proved; this can be said, however, that the first lock hospitals were called "lazar" or "leper" houses. An instance of this is the ancient hospital which stood at Kingsland (see illustration), which was called "Le Lokes." A "lock" was formerly used as a synonymous term with a lazar or poor-house, it being derived from "loques," an obsolete French word signifying rags. About the time of the foundation of the Kingsland institution England, with the rest of Europe, was being ravaged by an unrecognised scourge, which was afterwards found to be syphilis. To the master and governors of "Le Lokes" in the year 1437 was left by John Pope, citizen of London, a rent charge of 6s. 8d. issuing out of certain houses in the City.

This hospital is recorded to have been an appendage to St. Bartholomew's, but how and when it became annexed to it does not appear upon record. It was used as a kind of outer ward till the year 1761, when all the patients (according to the journals of St. Bartholomew's Hospital) were removed from Kingsland and the site of the hospital was let on a building lease. The neighbouring inhabitants having petitioned that the chapel might continue, and that service might be performed there, it was repaired, and, according to Lysons' "Environs of London," was still used in 1795. The same authority says that the architecture is Gothic and the building very small, which points will be seen to agree with our illustration. Another authority states that the structure was burned down in the eighteenth century, ultimately rebuilt, and then pulled down in 1846.

LOCK HOSPITAL AND CHAPEL, KINGSLAND, ABOUT 1780 (*from an old print*).

There was another hospital called the "Locke" at St. George's, Southwark, this probably being the one shown in old maps. This institution was for leprous persons as far back as the time of Edward II. Some dreary swamps in the neighbourhood were called Lock Fields. Another hospital for venereal diseases, called the Hospital

Previous articles appeared on Nov. 13, and Dec. 4.

**The History of Hospitals**—(*continued*).

Misericordia, existed in Goodman's Fields, near the Minories, in the year 1805.

### The London Lock Hospital.

The natural successor of all these institutions is the London Lock Hospital, founded in 1746, now the only institution of its kind in London, with branches in Harrow Road (for women) and Dean Street, Soho (for men). I am indebted to the Secretary, Mr. H. J. Eason, for some of the foregoing information, and for an interesting account of his own hospital.

This well-known institution was founded in the year 1746, at the instance of Mr. William Bromfeild, surgeon to the Prince of Wales and to St. George's Hospital, there being no provision made for venereal cases in general hospitals. The leasehold of a house and grounds was procured, described as "on the west side of the King's High Way leading from Hyde Park Corner to the King's private road to Chelsea" (now known as Grosvenor Place), not far from St. George's Hospital, which then stood at Hyde Park Corner. For the house was paid £350, with an annual ground rent of £19. The first matron had a salary of £15, and the nurses were paid £6 per annum, with a gratuity of £5 or £4 at Christmas if they behaved themselves.

The Old Lock Hospital, Hyde Park Corner, about 1840 (*from an old lithograph*).

The hospital was opened on January 31, 1747, with thirty beds. The first annual report is to be found at the British Museum. It is a curious old folio, headed by a cut of what seems to have been the proposed permanent building. It states that between January 31, 1747, and September 1749, of the 695 patients received, 644 had been discharged cured. A number of influential people gave their support, including several artistic friends, such as David Garrick, who gave a benefit play at Drury Lane, and Handel, who arranged an oratorio at the Opera House. Amongst other actors and musical people who lent their aid may be mentioned J. Rich, J. Lacey, John Board, Giardini, Linley, Barry, Foote, Powell, and Martin. In 1761 "The Firework" at Ranelagh produced £300 for the charity.

### An Unstable Benefactress.

In 1754 the new building was ready, and the hospital may be said to have fairly started. About this time it was reported at the board meeting that a lady of quality, hearing of the resolution of the governors to fit up a ward for married women only, had generously paid the expense thereof, a precedent noted to be worthy to be followed by every humane person. It is sad to relate that some years later (1772) it was discovered that this lady had lost her fortune and was selling her admission order to the hospital, which she held as a governor. The board made a bargain, in view of her former services, that if she would relinquish her rights as a governor they would grant her a pension of two shillings and sixpence per week.

During enlargement in 1766 the patients were accommodated on the ground floor. Complaints were made on the one hand about the patients being allowed to stand about the doorsteps, and on the other of the number of improper persons who visited the wards, bringing in intoxicants and endeavouring to persuade inmates to return to their former evil ways when discharged. The move upstairs again quickly put these matters right, and discipline was improved by providing the messenger with a constable's staff.

### The Dispensary Department.

The grounds were improved after some trouble with the Turnpike Trustees concerning a dispute as to whose duty it was to fill up a ditch adjacent to the hospital. In 1779 we find that the garden was used not merely for recreation. In that year a sub-committee was appointed to inquire into the great expense of drugs, who advised that balm and sage for the patients should be raised in the garden. The old diet table shows that before the days of teas, cocoa, and what we now consider to be necessaries, the alternative drink to milk or gruel was balm or sage tea.

In 1758 the Rev. Mr. Madan accepted the post of honorary chaplain, and the Lock chapel was built in 1764 and brought in a steady income to the funds of the hospital, the pew rental producing about £1,000 a year. Mr. Madan succeeded a gentleman who had been appointed as "divine" at the foundation, later to be dismissed for soliciting subscriptions on his own account.

About 1790 an asylum for female patients was opened in Osnaburg Row, an adjoining small street, and thus the principle of the separate buildings for men and women was established. This asylum was moved in 1812 to a new position in Knightsbridge, opposite the spot where the Hyde Park Hotel now stands. Concurrently with the service of the asylum efficient rescue work was carried out.

The end of the ninety-four years' lease of the ground in Grosvenor Place was reached in 1841, and the Marquis of Westminster, having refused an extension, it was arranged to move the hospital, asylum, and chapel to Westbourne Green, facing the Harrow Road. The history of that excellent institution the London Lock Hospital and Rescue Home on its present site, and at its branch hospital in Dean Street, Soho, is too well known for mention in an article which chiefly deals with hospitals in long-bygone years.

## Figures and Tables

**Figure f1:**
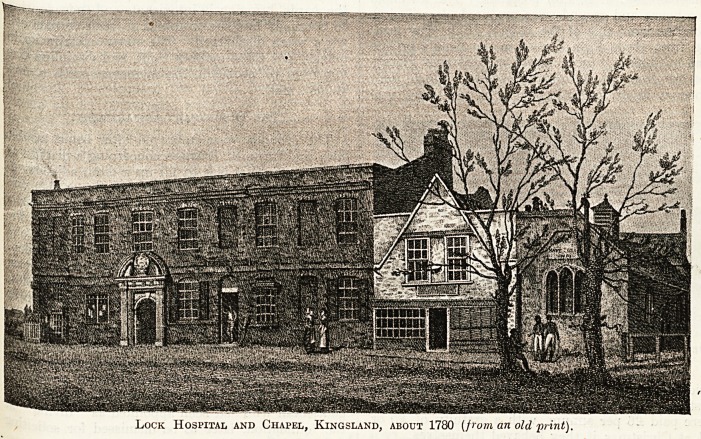


**Figure f2:**